# Alcohol Consumption Moderated the Association Between Levels of High Blood Lead or Total Urinary Arsenic and Bone Loss

**DOI:** 10.3389/fendo.2021.782174

**Published:** 2021-12-03

**Authors:** Yu-Mei Hsueh, Ya-Li Huang, Hsi-Hsien Chen, Horng-Sheng Shiue, Ying-Chin Lin, Ru-Lan Hsieh

**Affiliations:** ^1^ Department of Family Medicine, Wan Fang Hospital, Taipei Medical University, Taipei, Taiwan; ^2^ Department of Public Health, School of Medicine, College of Medicine, Taipei Medical University, Taipei, Taiwan; ^3^ Division of Nephrology, Department of Internal Medicine, School of Medicine, College of Medicine, Taipei Medical University, Taipei, Taiwan; ^4^ Division of Nephrology, Department of Internal Medicine, Taipei Medical University Hospital, Taipei, Taiwan; ^5^ Department of Chinese Medicine, Chang Gung University College of Medicine, Taoyuan, Taiwan; ^6^ Department of Family Medicine, School of Medicine, College of Medicine, Taipei Medical University, Taipei, Taiwan; ^7^ Department of Geriatric Medicine, School of Medicine, College of Medicine, Taipei Medical University, Taipei, Taiwan; ^8^ Department of Physical Medicine and Rehabilitation, Shin Kong Wu Ho-Su Memorial Hospital, Taipei, Taiwan; ^9^ Department of Physical Medicine and Rehabilitation, School of Medicine, College of Medicine, Taipei Medical University, Taipei, Taiwan

**Keywords:** alcohol, coffee, arsenic, lead, bone mineral density

## Abstract

Metal exposure and lifestyle are important risk factors for osteoporosis. Our study aimed to investigate the association between red blood cell lead and cadmium, total urinary arsenic, and plasma selenium levels and bone mineral density (BMD). In addition, we explored whether alcohol and coffee consumption modified the association between BMD and metals and metalloids. In total, 437 participants who underwent adult or senile physical examinations were recruited. Bone loss was defined as a calcaneus BMD T-score of <-1. Blood cadmium and lead and plasma selenium levels were measured using inductively coupled plasma mass spectrometry. Levels of urinary arsenic species were determined using high-performance liquid chromatography–hydride generator–atomic absorption spectrometry. The total urinary arsenic level was defined as the sum of the levels of urinary arsenic species. The BMD T-scores decreased significantly with increasing blood lead levels. The BMD T-scores also showed a downward trend with increasing total urinary arsenic levels. The odds ratio (OR) and 95% confidence interval (CI) for bone loss in patients with blood lead levels >57.58 *versus* 35.74 μg/dL were 1.98 and 1.17–3.34. In addition, the greater the lead or arsenic exposure and alcohol intake was the higher the OR for bone loss with multivariate ORs of 2.57 (95% CI 1.45–4.56) and 2.96 (95% CI 1.67–5.22), respectively. To the best of our knowledge, this study is the first to demonstrate that high total urinary arsenic or blood lead levels and frequent or occasional alcohol consumption had a significant multiplicative interaction for increasing the OR for bone loss.

## Introduction

Population aging is a global phenomenon (1). The bone structure constantly undergoes remodeling, and bone mass and density decrease with aging (2). Aging causes a redistribution of visceral fat mass (3), leads to development of osteosarcopenic obesity (4), and reduces height and bone mineral density (BMD). Therefore, osteoporosis is an important healthcare problem for the aging population.

According to the 1999–2001 Taiwan Health Insurance data, the average prevalence of osteoporosis in people aged ≥50 years was 1.63% for men and 11.35% for women (5). The World Health Organization (WHO) recommends using dual-energy X-ray absorptiometry to measure BMD in the spine, hip, or forearm for diagnosing osteoporosis (6). According to the WHO standards, normal BMD is defined as a BMD T-score of ≥-1. A BMD T-score between -1.0 and -2.5 indicates osteopenia, while a BMD T-score of ≤-2.5 indicates osteoporosis (6). According to recent studies, the important risk factors for osteoporosis include demographic variables (e.g., female sex, old age, menopausal age, and low body mass index), lifestyle (e.g., alcohol consumption and nutrition), and disease history (e.g., pregnancy disease, glucocorticoid use, previous fractures, rheumatoid arthritis, hyperthyroidism, hypogonadism, and chronic liver disease) (7). The existence of other risk factors is currently being investigated.

The bone is an important target organ for heavy metal toxicity. A significant correlation between blood lead and cadmium concentrations and low bone density has been observed in South Korean adults, suggesting that heavy metal exposure may be a risk factor for osteoporosis (8). However, in a previous study, the dietary intake of cadmium and lead by postmenopausal women in Spain did not affect bone health (9). Lead is a toxic substance. A Taiwanese study found that adults, especially women, had high urinary lead levels, which might lead to increased bone loss and osteoporosis (10). Another study demonstrated that low bone density was significantly associated with increased blood lead and cadmium and urinary lead levels in areas exposed to heavy metals in Guizhou, China (11). However, in a previous study, dietary intake of lead and cadmium by healthy women before menopause in Caceres did not affect bone health; it is likely that dietary exposure levels for cadmium and lead are mostly within the recommended range (12). Thus, the relationship between lead and cadmium exposure and BMD is unclear and warrants further investigation.

In 1987, inorganic arsenic was confirmed as a carcinogen in human skin and lung cancer (13). Epidemiological studies have shown that chronic arsenic exposure is associated with liver, bladder, kidney, and prostate cancer; hypertension; diabetes; and cardiovascular disease (14–16). An occupation exposure study showed that arsenic levels in hair are related to bone metabolism and bone loss in men in Turkey (17). Furthermore, administration of 0.05 and 0.5 ppm of inorganic arsenic to rats in drinking water for 12 weeks changed the bone microstructure and mineral density (18). Although there is a wide range of arsenic exposure worldwide, few epidemiological studies have evaluated the association between chronic arsenic exposure and low bone density.

Selenium is an important antioxidant, and there are many types of selenoproteins, including antioxidant enzymes or enzyme coenzymes, in the body (19). In healthy humans, selenium levels are negatively correlated with bone metabolism and positively correlated with bone density (20). One study found that elderly patients with disability had low selenium and zinc levels, impaired total antioxidant capacity, and low bone density (21). However, another study failed to demonstrate a correlation between serum selenium levels and bone mass (22). Thus, the correlation between selenium levels and bone density is controversial.

Alcohol consumption and coffee consumption are well-known lifestyle factors. Regular or frequent alcohol consumption is considered to affect BMD (23). Drinking coffee is considered harmful to bone metabolism as caffeine intake affects calcium absorption (24). However, coffee also contains some physiologically beneficial ingredients (25). However, it is unclear whether alcohol or coffee consumption modifies the effects of lead, cadmium, arsenic, and selenium on BMD. However, studies have revealed that selenium plays a protective role against oxidative damage caused by arsenic, cadmium, and lead (26, 27). Therefore, we aimed to explore the association between bone loss and red blood cell lead and cadmium, total urinary arsenic, and plasma selenium levels among people participating in adult and senile health examinations in areas without environmental heavy metal pollution. In addition, we investigated the effects of the interactions between lifestyle (e.g., alcohol and coffee consumption) and red blood cell lead and cadmium, total urinary arsenic, and plasma selenium levels on bone loss.

## Materials and Methods

### Study Subjects, Interviews, and Specimen Collection

Employees aged 40–60 years in public and private organizations undergo an annual health examination, which is a benefit provided to employees in Taiwan. They can go to designated hospitals for health examinations. The research assistant explained the purpose of the research during the health examinations. Participants signed informed consent voluntarily before study commencement. We recruited 387 subjects who underwent adult health examinations and 50 subjects who underwent senile health examinations at the Health Center of Wanfang Hospital between July 2007 and September 2011 (28). All participants lived in Taipei City, where there are no factories causing environmental metal pollution. All study subjects were interviewed, and their blood and urine samples were collected as previously described (28). The Research Ethics Committee of Taipei Medical University, Taiwan (TMU-Joint Institutional Review Board N202007046) approved this study, which was conducted in accordance with the Declaration of Helsinki.

### Bone Mineral Density Measurements

BMD in the calcaneus was measured using quantitative ultrasound to obtain broadband ultrasound attenuation. Ultrasound passes through the bone tissue at different speeds simultaneously, showing different attenuations. Quantitative ultrasound is a convenient and affordable tool for assessing BMD. The results for each participant were compared with the ideal or peak BMD of a healthy 30-year-old adult to obtain the T-score. According to the WHO standards, a T-score of ≤-2.5 indicates osteoporosis, T score between 1.0 and -2.5 represents low BMD (osteopenia), and a T score of ≥-1 is considered normal BMD (6). Because there were very few participants with a T-score of ≤-2.5, patients with a T-score of ≤-2.5 and between -1 and -2.5 were collectively considered to have bone loss (29).

### Urinary Arsenic Species Measurements

The measurements of arsenite (As^III^), arsenate (As^V^), monomethylarsonic acid (MMA^V^), and dimethylarsinic acid (DMA^V^) in the urine have been described previously (30). **Table 1** shows the measurement methods, detection limits, standard reference materials, and recovery rates. The detection limits of As^III^, As^V^, MMA^V^, and DMA^V^ were 0.02, 0.10, 0.07 and 0.06 (μg/L) respectively. The accuracy and reliability of the results were evaluated by standard reference materials (meet the certificate value), recovery rate (93.8–102.2%) and coefficient of variance (< 10%). The total urinary arsenic level (μg/g creatinine) was the sum of the As^III^, As^V^, MMA^V^, and DMA^V^ levels represents the arsenic exposure, which was divided by the urinary creatinine level to control for hydration. The measurement of creatinine levels is described in **Table 1**.

**Table 1:** Validity and reliability of the measurements for determining urinary arsenic species, red blood cell lead and cadmium, and plasma selenium concentrations.

Metals or metalloids	Instrument	Detection limit (μg/L)	Recovery rate	Standard reference materials (SRM)	Coefficient of variance (CV)
Red blood cell lead	Inductively coupled plasma-mass spectrometry (ICP-MS)	0.32	80 - 120%	Seronorm Trace Elements Whole Blood L-2 (Lot 1103129); certificate value 310.0 μg/L (range 186.0−434.0 μg/L); 329.0 ± 17.0 μg/L in our system	<5%
Red blood cell cadmium	ICP-MS	0.07	80 - 120%	Seronorm Trace Elements Whole Blood L-2 (Lot 1103129); certificate value 5.8 μg/L (range 5.4−6.2 μg/L); 6.1 ± 0.5 μg/L in our system	<5%
Arsenite (As^III^)	High-performance liquid chromatography-hydride generator-atomic absorption spectrometry	0.02	93.8–102.2%	National Institute of Standards and Technology 2670 (NIST, Gaithersburg, MD, USA); certificate value 480 ± 100 μg/L inorganic arsenic; 507 ± 17 μg/L in our system (n = 4)	<10%
Arsenate (As^V^)		0.10			
Monomethylarsonic acid (MMA^V^)		0.07			
Dimethylarsinic acid (DMA^V^)		0.06			
Urinary creatinine level	Roche Modular P800 instrument (colorimetric assay to measure creatinine-picric acid complex formation)				
Plasma selenium	ICP-MS	0.193	80 - 120%	Seronorm Trace Elements Whole Blood Label II (SERO AS, Norway); certificate value 112 ± 46 mg/L selenium; 118.7 ± 11.1 mg/L in our system (n = 7)	9.8%

### Determination of Lead and Cadmium Levels in Red Blood Cells and Selenium Levels in the Plasma

Red blood cell lead and cadmium levels were measured using inductively coupled plasma mass spectrometry (ICP-MS) (31). The detection limits, validity, and reliability of the measurements are presented in **Table 1**. The measurement of plasma selenium levels was performed using ICP-MS (32). The accuracy and reliability of the results were evaluated by standard reference materials (meet the certificate value), recovery rate (80.0–120.0%) and coefficient of variance (< 5%). The detection limit, recovery rate, and standard reference materials are listed in **Table 1**.

### Statistical Analysis

Continuous variables are presented as mean ± standard deviation. The Kruskal–Wallis test was used to compare continuous variables between more than two groups, and the Wilcoxon rank-sum test was used to compare the differences in the continuous variables between two groups. A multivariate linear regression model was used to determine the correlation between the BMD T-scores and red blood cell cadmium and lead, total urinary arsenic, and plasma selenium levels after adjusting for confounders. Multiple logistic regression analysis was used to assess the association between the potential risk factors and bone loss. For these analyses, the cutoff points for continuous variables among independent variables were the corresponding tertiles of the reference group. The multivariate-adjusted odds ratio (OR) and 95% confidence interval (CI) were used to calculate the risk of bone loss. OR is the ratio of the exposure odds in cases to the exposure odds in controls. It shows the relationship between exposure and disease; OR >1 indicates that exposure is a risk factor for diseases, and OR <1 indicates that exposure is a protective factor for diseases. The significance test for a linear trend of the OR in the exposed stratification used categorized exposure variables as score variables, which performed as continuous variables. The cutoff points for the risk factors in the interaction analysis were the medians of the reference group. The additive interactions between the risk factors on the OR for bone loss were evaluated in a pairwise manner using the synergy index provided by Rothman (33). The observed synergy index value was not equal to 1, indicating an additive interaction, and the OR values and their variance–covariance matrix were used to calculate the 95% CI (34). The product term between the red blood cell lead or total urinary arsenic levels and alcohol and coffee consumption were used pairwise to test the multiplicative interaction on the OR for bone loss in the multiple logistic regression model. SAS package (version 9.4; SAS Institute, Cary, NC) was used for all data analyses. Statistical significance was set at *p* < 0.05.

## Results

The sociodemographic characteristics, lifestyles, disease histories, and BMD T-scores are presented in **Supplementary Table S1**. The median BMD T-score for the 437 participants was -0.8 (range, -3.5– 3.5). The mean BMD T-score was -0.78 ± 1.08. Lower median BMD T-scores were associated with older age. The BMD T-scores of women were significantly lower than those of men. In addition, the median BMD T-score increased significantly with increasing educational level. Moreover, frequent or occasional coffee drinkers had significantly higher BMD T-scores than non-drinkers. There were no differences in the BMD T-scores between cigarette smokers and non-smokers, the three groups of tea and alcohol consumption, those with and without diabetes, and those with and without hypertension.

In total, 437 people participated in the study. The average age and standard deviation of normal BMD were 52.05 and 10.12 years, respectively, which was significantly lower than those of bone loss (55.87 and 9.65 years, respectively). The OR for bone loss significantly increased in a dose–response manner with the increasing age. The OR for bone loss in women was significantly higher than that in men. Frequent or occasional alcohol drinkers had a significantly higher OR (1.61, 95% CI 1.04–2.50) for bone loss than non-drinkers. The OR for bone loss in occasional or frequent coffee drinkers (0.61, 95% CI 0.41–0.90) was significantly lower than that in non-drinkers (**Table 2**). Regarding educational status, a higher education level was associated with a lower the risk of bone loss, but there was not significant after adjusting for age and sex. Similarly, tea consumption, body mass index, and disease histories were not related to bone loss. There were few smokers in this study, and cumulative cigarette smoking (pack-years) was not associated with bone loss (data not shown).

**Table 2:** The association between sociodemographic characteristics, lifestyle, and disease history and bone loss.

Variables	Bone loss N (%)	Normal bone mineral density N (%)	Age-genderadjusted OR (95% CI)
Age (years)	55.87 ± 9.65^#,***^	52.05 ± 10.12^#,***^	
<50	38 (20.99)	102 (39.84)	1.00 ^a,§,***^
50–65	116 (64.09)	131 (51.17)	2.18 (1.38-3.45) ^***^
>65	27 (14.92)	23 (8.98)	2.98 (1.52-5.84) ^***^
Sex			
Male	102 (56.35)	177 (69.14)	1.00 ^b^
Female	79 (43.65)	79 (30.86)	1.58 (1.06-2.37) ^*^
BMI (kg/m^2^)			
24	114 (62.98)	141 (55.08)	1.00
24–27	36 (19.89)	64 (25.00)	0.64 (0.39-1.05)^+^
27	31 (17.13)	51 (19.92)	0.73 (0.43-1.23)
Educational level			
Illiterate/elementary	36 (19.89)	26 (10.16)	1.00 ^§,*^
Junior/senior high	56 (30.94)	73 (28.52)	0.76 (0.40-1.45)
College and above	89 (49.17)	157 (61.33)	0.61 (0.33-1.13)
Cigarette smoking			
Non-smoker	133 (73.48)	175 (68.63)	1.00
Smoker	48 (26.52)	81 (31.37)	1.00 (0.63-1.61)
Alcohol consumption			
Never	108 (59.67)	160 (62.50)	1.00 ^§,**^
Frequent	29 (16.02)	50 (19.33)	1.23 (0.70 - 2.15)
Occasional	44 (24.31)	46 (17.97)	2.02 (1.20 - 3.41)^**^
Frequent or occasional	73 (40.33)	96 (37.50)	1.61 (1.04-2.50) ^*^
Coffee consumption			
No	98 (54.14)	107 (41.80)	1.00
Frequent	46 (25.41)	95 (37.11)	0.52 (0.33- 0.82) ^**^
Occasional	37 (20.44)	54 (21.09)	0.77 (0.46 - 1.29)
Frequent or occasional	83 (45.86)	149 (58.20)	0.61 (0.41- 0.90)^*^
Tea consumption			
No	72 (39.78)	82 (31.64)	1.00
Frequent	68 (37.57)	132 (51.56)	0.63 (0.41 - 0.99) ^*^
Occasional	41 (22.65)	43 (16.80)	1.24 (0.72 - 2.16)
Frequent or occasional	109 (60.22)	175 (68.36)	0.78 (0.52- 1.18)
Diabetes			
No	168 (92.82)	238 (92.97)	1.00
Yes	13 (7.18)	18 (7.03)	0.83 (0.38-1.78)
Hypertension			
No	144 (79.56)	201 (79.45)	1.00
Yes	37 (20.44)	52 (20.55)	0.83 (0.51-1.36)

OR, odds ratio; CI, confidence interval; BMI, body mass index.

Values are expressed as the mean ± standard deviation.

^#^Wilcoxon rank-sum test was tested for age between bone loss group and normal bone mineral density group.

^a^OR adjusted for sex. ^b^OR adjusted for age. ^§^Test for trend-dose response relationship.

^+^0.05 < p < 0.1, ^*^p < 0.05, **p < 0.01, ^***^p < 0.001.

**Figure 1:**
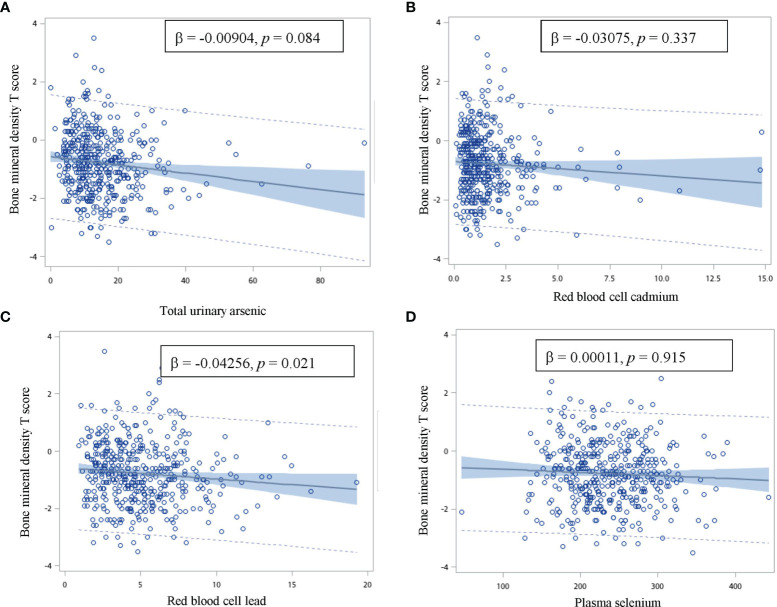
Correlation between the bone mineral density T score and total urinary arsenic, red blood cell cadmium and lead, and plasma selenium concentration. (β: age, gender, and alcohol and coffee consumption adjusted regression coefficient). The vertical axis is the bone mineral density T score, and the horizontal axis is **(A)** total urinary arsenic **(B)** red blood cell cadmium **(C)** red blood cell lead, and **(D)** plasma selenium.

The correlations between the BMD T-scores and total urinary arsenic, red blood cell lead and cadmium, and plasma selenium levels are presented in **Figure 1**. As the red blood cell lead levels increased, the BMD T-scores significantly decreased after adjusting for age, sex, and alcohol and coffee consumption (regression coefficient = -0.04256; *p* = 0.021). The BMD T-scores also tended to decrease with increasing total urinary arsenic concentrations after adjusting for age, sex, and alcohol and coffee consumption. No relationships were observed between the BMD T-scores and red blood cell cadmium or plasma selenium levels.

The OR for bone loss increased significantly when the red blood cell lead level increased (**Table 3**), showing a dose–response relationship after adjusting for age, sex, and alcohol and coffee consumption. The OR for bone loss with red blood cell lead level >57.58 *versus* ≤35.74 μg/dL was 1.98 (95% CI 1.17–3.34) after multivariate adjustment. The total urinary arsenic level was positively correlated with the OR for bone loss; however, red blood cell cadmium and plasma selenium levels were not associated with the OR for bone loss. However, arsenic species (As^III^, As^V^, MMA^V^, and DMA^V^) did not differ between the normal bone group and the bone loss group (data not shown).

**Table 3:** The association between total urinary arsenic, red blood cell lead and cadmium, and plasma selenium levels and bone loss.

Variables	Bone loss N (%)	Normal bone mineral densityN (%)	Age-gender adjusted OR (95% CI)	Multivariate Adjusted OR (95% CI)
Total urinary arsenic (μg/g creatinine)	15.92 ± 9.01^a,**^	13.95 ± 10.22 ^a,**^		
≤9.38	42 (23.2)	85 (33.20)	1.00 ^§,*^	1.00^§,+^
>9.38–14.71	55 (30.39)	86 (33.59)	1.20 (0.72 – 2.00)	1.08 (0.64 – 1.82)
>14.71	84 (46.41)	85 (33.20)	1.76 (1.08 – 2.87) ^*^	1.60 (0.97 – 2.63)^+^
Red blood cell lead (μg/dL)	53.81 ± 28.55 ^a *^	48.10 ± 26.13 ^a *^		
≤35.74	38 (20.99)	86 (33.59)	1.00^§,**^	1.00^§,*^
>35.74–57.58	73 (40.33)	85 (33.20)	2.02 (1.22 – 3.35) ^**^	1.96 (1.17 – 3.28) ^*^
>57.58	70 (38.67)	85 (33.20)	2.11 (1.27 – 3.53) ^**^	1.98 (1.17 – 3.34) ^*^
Red blood cell cadmium (μg/L)	1.62 ± 1.46	1.56 ± 1.66		
≤0.84	55 (30.39)	88 (34.38)	1.00	1.00
>0.84–1.60	59 (32.60)	83 (32.42)	0.99 (0.60 – 1.63)	0.91 (0.55 – 1.52)
>1.60	67 (37.02)	85 (33.20)	1.11 (0.68 – 1.80)	0.92 (0.56 – 1.53)
Plasma selenium (μg/L)	237.86 ± 54.57	235.82 ± 49.54		
≤211.4	54 (29.83)	86 (33.59)	1.00	1.00
>211.4–258.0	70 (38.67)	85 (33.20)	1.09 (0.67 – 1.76)	1.14 (0.70 – 1.85)
>258.0	57 (31.49)	85 (33.20)	0.82 (0.49 – 1.35)	0.87 (0.52 – 1.44)

OR, odds ratio; CI, confidence interval.

Values are expressed as the mean ± standard deviation.

Multivariate-adjusted OR was adjusted for age, gender, alcohol, and coffee consumption.

a Wilcoxon rank-sum test was tested for total urinary arsenic and red blood cell lead concentration between bone loss group and normal bone mineral density group.

^§^Test for trend -dose response relationship.

^+^0.05 < p < 0.1, ^*^p < 0.05, ^**^p < 0.01.

In our study, the red blood cell lead level was significantly higher and the plasma selenium level was significantly lower in frequent or occasional alcohol drinkers than in non-drinkers. The levels of total urinary arsenic, red blood cell lead and cadmium, and plasma selenium levels did not differ according to coffee consumption status (**Supplementary Table S2**).

Because alcohol and coffee consumption, red blood cell lead levels, and total urinary arsenic levels were related to the OR for bone loss, we further analyzed the effects of the interactions between red blood cell lead levels, total urinary arsenic levels, and alcohol or coffee consumption on the OR for bone loss (**Table 4**). When examining the risk factors of total urinary arsenic levels >11.35 μg/g creatinine and frequent or occasional alcohol consumption, the OR for bone loss gradually increased in a dose-dependent manner (i.e., from exposure to neither risk factor, one risk factor, and both risk factors). Participants with total urinary arsenic levels >11.35 μg/g creatinine and frequent or occasional alcohol consumption had an OR for bone loss of 2.57 (95% CI 1.45–4.56) compared to those participants who never consumed alcohol and had a total urinary arsenic level of ≤11.35 μg/g creatinine. A similar phenomenon was observed for the combined effect of high total urinary arsenic levels and non-consumption of coffee; high red blood cell lead levels and frequent or occasional alcohol consumption and/or never consumed coffee; high total urinary arsenic levels and high red blood cell lead levels; and non-consumption of coffee and frequent or occasional alcohol consumption. In addition, high total urinary arsenic or high red blood cell lead levels and frequent or occasional alcohol consumption had a significant multiplicative interaction for increasing the OR for bone loss (**Table 4**).

**Table 4:** The combined effect of alcohol or coffee consumption and total urinary arsenic or red blood cell lead levels on bone loss.

Variable 1	Variable 2	Bone loss Yes/No	Age-sex adjusted OR (95% CI)	Multivariate-adjusted OR (95% CI)
Total urinary arsenic (μg/g creatinine)	Alcohol consumption			
≤11.35	Never	40/85	1.00 ^§,**^	1.00 ^§,**^
≤11.35	Occasional or frequent	23/43	1.49 (0.77 – 2.90)	1.61 (0.82 – 3.15)
>11.35	Never	68/75	1.62 (0.97 – 2.72)^+^	1.60 (0.95 – 2.69) ^+^
>11.35	Occasional or frequent	50/53	2.49 (1.41 – 4.40) ^**^	2.57 (1.45 – 4.56) ^**^
Synergy index			1.34 (0.42 – 4.30)	1.30 (0.42 – 4.01)
P _interaction_			0.025	0.016
Total urinary arsenic (μg/g creatinine)	Coffee consumption			
≤11.35	Occasional or frequent	29/78	1.00 ^§,***^	1.00 ^§,**^
≤11.35	Never	34/50	1.81 (0.97 – 3.39)^+^	1.92 (1.02 – 3.60) ^*^
>11.35	Occasional or frequent	54/71	1.85 (1.05 – 3.25) ^*^	1.78 (1.01 – 3.13) ^*^
>11.35	Never	64/57	2.77 (1.57 – 4.88) ^*^	2.75 (1.55 – 4.86) ^***^
Synergy index			1.07 (0.44 – 2.61)	1.03 (0.42 – 2.52)
P _interaction_			0.682	0.495
Red blood cell lead (μg/L)	Alcohol consumption			
≤42.23	Never	46/92	1.00 ^§,***^	1.00 ^§,***^
≤42.23	Occasional or frequent	19/36	1.57 (0.78 – 3.16)	1.69 (0.83 – 3.44)
>42.23	Never	62/68	2.02 (1.21 – 3.39) ^**^	2.08 (1.24 – 3.49) ^**^
>42.23	Occasional or frequent	54/60	2.75 (1.57 – 4.80) ^***^	2.96 (1.67 – 5.22) ^***^
Synergy index			1.10 (0.42 – 2.90)	1.11 (0.44 – 2.81)
P _interaction_			0.025	0.018
Red blood cell lead (μg/L)	Coffee consumption			
≤42.23	Occasional or frequent	29/71	1.00 ^§,***^	1.00 ^§,***^
≤42.23	Never	36/57	1.53 (0.82 – 2.84)	1.59 (0.86 – 2.97)
>42.23	Occasional or frequent	54/78	1.90 (1.08 – 3.36) ^*^	1.80 (1.01 – 3.19) ^*^
>42.23	Never	62/50	3.48 (1.92 – 6.29) ^***^	3.39 (1.87 – 6.15) ^***^
Synergy index			1.73 (0.66 – 4.53)	1.72 (0.64 – 4.59)
P _interaction_			0.547	0.410
Total urinary arsenic (μg/g creatinine)	Red blood cell lead (μg/L)			
≤11.35	≤ 42.23	20/68	1.00 ^§,***^	1.00 ^§,***^
>11.35	≤ 42.23	45/60	2.49 (1.22 – 5.11) ^*^	2.28 (1.10 – 4.73) ^*^
≤11.35	> 42.23	43/60	2.87 (1.34 – 6.15) ^**^	2.77 (1.27 – 6.04) ^*^
>11.35	> 42.23	73/68	4.42 (1.94 – 10.11) ^***^	4.12 (1.77 – 9.58) ^***^
Synergy index			1.02 (0.50 – 2.08)	1.02 (0.49 -2.15)
P _interaction_			0.062	0.166
Alcohol consumption	Coffee consumption			
Never	Occasional or frequent	47/88	1.00 ^§,**^	1.00 ^§,**^
Occasional or frequent	Occasional or frequent	36/61	1.65 (0.92 – 2.98)^+^	1.56 (0.86 – 2.84)
Never	Never	61/72	1.68 (1.00 – 2.81) ^*^	1.68 (1.00 – 2.82) ^*^
Occasional or frequent	Never	37/35	2.97 (1.57 – 5.62) ^***^	2.74 (1.44 – 5.22) ^**^
Synergy index			1.48 (0.52 – 4.25)	1.40 (0.46 – 4.25)
P _interaction_			0.730	0.671

OR, odds ratio; CI, confidence interval.

Multivariate-adjusted OR was adjusted for age, gender, and alcohol or coffee consumption.

^§^Test for tend -dose response relationship.

^+^0.05< p < 0.1, ^*^p < 0.05, ^**^p < 0.01, ^***^p < 0.001.

## Discussion

We found that age and sex influenced BMD T-scores. Alcohol consumption increased the OR for bone loss, while coffee consumption reduced the OR. An increase in total urinary arsenic or red blood cell lead levels also increased the OR for bone loss. In addition, to the best of our knowledge, this study is the first observational study to report that high total urinary arsenic or red blood cell lead levels and frequent or occasional alcohol consumption had a significant multiplicative interaction that increased the OR for bone loss.

The study participants had no occupational exposure, but they may have been exposed to arsenic, lead, and cadmium from contaminated rice, poultry and livestock meat, and seafood (35–37).Exposure to toxic metals and metalloids is a risk factor for fractures and degenerative bone diseases (38). High total arsenic levels in the urine can also cause BMD loss (29). In this study, we found that increased total urinary arsenic levels were related to the OR for BMD loss, possibly because exposure to arsenic increases fasting blood sugar (39). Abnormal glucose metabolism causes systemic inflammation, which may be related to the activation of bone resorption, leading to decreased BMD (40, 41).

As lead accumulation in the body increases, bone density decreases and the risk of fractures increases (42). Experiments in male mice showed that long-term exposure to low lead concentrations caused a decline in bone formation and damage to the bone microstructure (43). In addition, there is a significant correlation between low blood lead levels and the prevalence of osteoporosis, especially among smokers in South Korea (44). In this study, we found that increased red blood cell lead concentration was associated with a significant increase in the OR for bone loss. This finding may be explained by the fact that lead is stored in bone and interferes with the metabolism of calcium and phosphorus, which in turn damages the mineralization of bone and reduces BMD (45), and increasing the fracture risk (46) requires further investigation.

It is well known that cadmium exposure can alter bone formation, reduce bone mineralization, and increase the risk of fractures and osteoporosis (47). However, red blood cell cadmium levels were not related to bone loss in the present study. Although research has indicated that selenium intake is associated with bone health (20) and plasma selenium is positively correlated with bone density (21, 48), one study showed that high selenium levels could cause bone fracture (49). However, the relationship between plasma selenium levels and BMD was not demonstrated in the present study.

Chronic alcohol consumption in rats increases alveolar bone resorption and causes osteopenia and bone loss (50). A cross-sectional study in South Korea also found that heavy alcohol consumption in men was associated with lower BMD, while consumption of light alcohol by women was associated with higher BMD (51). In contrast, a cross-sectional study in South Koreans showed that moderate alcohol intake increased the areal BMD (52). This effect may be caused by the phenols in the wine, which inhibit bone absorption and inflammation, resulting in enhanced bone formation (53). In the present study, frequent or occasional alcohol consumption increased the OR for BMD loss. This might be due to increased red blood cell lead levels (**Supplementary Table S2**) or formation of reactive oxygen species through alcohol metabolism, which could increase bone resorption (54, 55). Reduced bone formation eventually causes decreased BMD (55, 56).

The composition of coffee is complex, and coffee intake has been shown to increase the risk of osteoporosis (57). An epidemiological study reported that high coffee intake (≥4 cups/day) reduced bone density (58). Furthermore, the adverse effects of coffee or caffeine intake on calcium balance in one study could be explained by decreased calcium absorption and increased urinary and fecal calcium excretion (59). In contrast, a follow-up study with men and premenopausal women found that coffee consumption was associated with high BMD T-scores and reduced osteoporosis risk in Taiwan (60). Moreover, a South Korean study showed that moderate coffee consumption by postmenopausal women had a protective effect on bone health (61). Furthermore, research in Hong Kong revealed that habitual coffee intake was significantly positively correlated with BMD of the lumbar spine and femoral neck (52). In the current study, we found that frequent or occasional coffee consumption reduced the OR for bone loss. Currently, the relationship between coffee consumption and bone mineral density is unclear and needs to be further explored.

In this study, we found that frequent or occasional alcohol consumption significantly modified the OR for the effect of red blood cell lead levels on bone loss. Simultaneous exposure of male Wistar rats to alcohol and lead, compared to exposure to alcohol or lead alone, reduced bone formation markers (e.g., osteocalcin, procollagen-1, osteoprotegerin, and alkaline phosphatase) and increased resorption markers (e.g., telopeptides of collagen-1 and soluble receptor activator of nuclear factor-kB ligand), leading to bone demineralization (62). These effects were accompanied by the disruption of hormonal regulation of mineral metabolism and an imbalance between calcium and phosphate levels. Therefore, simultaneous exposure to lead and alcohol altered bone metabolism more seriously than either lead or alcohol alone. In the present study, we found a significant multiplicative interaction between frequent or occasional alcohol consumption and total urinary arsenic levels, which increased the OR for bone loss. Frequent or occasional alcohol consumption may induce generation of reactive oxygen species and increase bone resorption (55). Arsenic may also induce generation of reactive oxygen species to promote the differentiation of osteoclasts through p38 phosphorylation, resulting in bone loss (63).

Some limitations of this study must be considered when interpreting the results. This was a cross-sectional study. Therefore, the causal relationship between red blood cell lead or total urinary arsenic levels and bone loss could not be confirmed. Samples were collected only once to evaluate the levels of red blood cell cadmium and lead, total urinary arsenic, and plasma selenium. However, if all participants maintained a stable lifestyle and their metabolism was homeostatic, these measurements might be reliable. The sample size was small, and data on other factors that could potentially affect BMD were not available. However, this study found that high red blood cell lead and total urinary arsenic levels were associated with bone loss.

## Conclusions

Our study demonstrated that high red blood cell lead and total urinary arsenic levels significantly increased the OR for bone loss in a dose–response manner. In addition, to the best of our knowledge, this study is the first epidemiological study to demonstrate that high total urinary arsenic or red blood cell lead levels and frequent or occasional alcohol consumption had a significant multiplicative interaction for increasing the OR for bone loss. These findings showed that the greater the lead or arsenic exposure and alcohol intake, the higher the OR for bone loss.

## Data Availability Statement

The data that support the findings of this study are available on request from the corresponding author.

## Ethics Statement

The studies involving human participants were reviewed and approved by Taipei Medical University-Joint Institutional Review Board. The patients/participants provided their written informed consent to participate in this study.

## Author Contributions

HHC and HSS partly contributed to the conception and design of the work; RLH and YCL recruited the study subjects; YMH has done the experiment; YLH contributed to the statistical analysis and analyzed the data. YMH wrote the manuscript; YCL and RLH performed the study design and executed the whole research plan and wrote review. All authors contributed to the article and approved the submitted version.

## Funding

This study was supported by grants from the Taipei Medical University-Wanfang Hospital Research Project (109TMU-WFH-04) and Ministry of Science and Technology, Taiwan [MOST103-2314-B-038-021-MY2 (1-2), MOST103-2314-B-038-021-MY2 (2-2), MOST 105-2314-B-038-082, MOST 106-2314-B-038-066, MOST 107-2314-B-038-073, MOST 108-2314-B-038-089, MOST 109-2314-B-038-081, and MOST 109-2314-B-038-067].

## Conflict of Interest

The authors declare that the research was conducted in the absence of any commercial or financial relationships that could be construed as a potential conflict of interest.

## Publisher’s Note

All claims expressed in this article are solely those of the authors and do not necessarily represent those of their affiliated organizations, or those of the publisher, the editors and the reviewers. Any product that may be evaluated in this article, or claim that may be made by its manufacturer, is not guaranteed or endorsed by the publisher.
