# Supplementary material for: Alcohol Consumption Moderated the Association Between Levels of High Blood Lead or Total Urinary Arsenic and Bone Loss
Source: Front Endocrinol (Lausanne). 2021 Dec 3;12:782174. doi: 10.3389/fendo.2021.782174 (PMC8678633; doi:10.3389/fendo.2021.782174)
Supplement: Supplementary file 1 [file Table_1.docx]

Supplementary Tables

Supplementary Table S1 Bone mineral density T scores based on sociodemographic characteristics, lifestyle, and disease histories

| Variables |  | Bone mineral density T score | | | | |
| --- | --- | --- | --- | --- | --- | --- |
|  | N | Median | Minimum | Maximum | Mean | Standard deviation |
| Total subjects | 437 | -0.80 | -3.50 | 3.50 | -0.78 | 1.08 |
| Age (years) |  |  |  |  |  |  |
| < 50 | 140 | -0.40 ^a,b,***^ | -2.70 | 2.90 | -0.37 | 1.05 |
| 50–65 | 247 | -1.00 ^a^ | -3.50 | 3.50 | -0.96 | 1.03 |
| > 65 | 50 | -1.15 ^b^ | -3.20 | 1.60 | -1.02 | 1.03 |
| Sex |  |  |  |  |  |  |
| Male | 276 | -0.60 ^c,***^ | -3.20 | 2.90 | -0.64 | 1.05 |
| Female | 158 | -1.05 ^c^ | -3.50 | 3.50 | -1.02 | 1.11 |
| BMI (kg/m^2^) |  |  |  |  |  |  |
| 24 | 255 | -0.90 | -3.50 | 3.50 | -0.86 | 1.09 |
| 24–27 | 100 | -0.70 | -3.20 | 1.70 | -0.66 | 1.06 |
| 27 | 82 | -0.70 | -3.00 | 2.90 | -0.67 | 1.08 |
| Educational level |  |  |  |  |  |  |
| Illiterate/elementary | 62 | -1.30 ^d,e,***^ | -3.50 | 2.50 | -1.23 | 1.12 |
| Junior/senior high | 129 | -0.80 ^d^ | -3.20 | 3.50 | -0.70 | 1.15 |
| College and above | 246 | -0.70 ^e^ | -3.20 | 2.40 | -0.71 | 1.01 |
| Cigarette smoking |  |  |  |  |  |  |
| Non-smoker | 308 | -0.85 | -3.50 | 2.50 | -0.82 | 1.08 |
| Former or current smoker | 128 | -0.80 | -3.20 | 2.90 | -0.67 | 1.10 |
| Alcohol consumption |  |  |  |  |  |  |
| Never | 268 | -0.80 | -3.30 | 3.50 | -0.82 | 1.05 |
| Frequent | 79 | -0.80 | -3.50 | 2.90 | -0.61 | 1.10 |
| Occasional | 90 | -1.00 | -2.70 | 2.50 | -0.80 | 1.16 |
| Frequent or occasional | 169 | -0.90 | -3.50 | 2.90 | -0.71 | 1.14 |
| Coffee consumption |  |  |  |  |  |  |
| No | 205 | -1.00 ^f,+^ | -3.50 | 3.50 | -0.87 | 1.15 |
| Frequent | 141 | -0.70 | -3.20 | 2.90 | -0.62 | 1.02 |
| Occasional | 91 | -0.90 | -3.20 | 1.80 | -0.81 | 1.00 |
| Frequent or occasional | 232 | -0.80 ^f^ | -3.20 | 2.90 | -0.70 | 1.01 |
| Tea consumption |  |  |  |  |  |  |
| No | 153 | -0.90 | -3.30 | 3.50 | -0.91 | 1.09 |
| Frequent | 200 | -0.70 | -3.20 | 2.90 | -0.64 | 1.07 |
| Occasional | 84 | -1,00 | -3.50 | 2.40 | -0.89 | 1.08 |
| Frequent or occasional | 284 | -0.80 | -3.50 | 2.90 | -0.71 | 1.08 |
| Diabetes |  |  |  |  |  |  |
| No | 406 | -0.80 | -3.50 | 3.50 | -0.78 | 1.10 |
| Yes | 31 | -0.80 | -2.60 | 0.90 | -0.77 | 0.82 |
| Hypertension |  |  |  |  |  |  |
| No | 345 | -0.90 | -3.50 | 3.50 | -0.79 | 1.09 |
| Yes | 89 | -0.80 | -3.30 | 2.50 | -0.78 | 1.06 |

*Abbreviations*: BMI, body mass index.

The same superscripted letters indicate that there was a significant difference in the bone mineral density T scores between the two groups, which was verified using the Kruskal-Wallis test and Wilcoxon rank-sum test. ^+^ 0.05 < *p* < 0.1, ^***^*p* < 0.001

Supplementary Table S2 The levels of total urinary arsenic, red blood cell lead and cadmium, and plasma selenium levels in different alcohol or coffee consumption status

|  | Alcohol consumption | | Coffee consumption | |
| --- | --- | --- | --- | --- |
|  | Never (N=268) | Occasional or frequent (N=169) | Never (N=205) | Occasional or frequent (N=232) |
| Total urinary arsenic (μg/g creatinine) | 14.24 ± 9.71 | 15.59 ± 9.84 | 14.74 ± 9.02 | 14.79 ± 10.41 |
|  |  |  |  |  |
| Red blood cell lead (μg/L) | 48.39 ± 28.68 ^a,**^ | 53.76 ± 24.61 ^a,**^ | 51.37 ± 28.25 | 49.66 ± 26.42 |
|  |  |  |  |  |
| Red blood cell cadmium (μg/L) | 1.54 ± 1.55 | 1.66 ± 1.63 | 1.58 ± 1.23 | 1.59 ± 1.83 |
|  |  |  |  |  |
| Plasma selenium (μg/L) | 241.9 ± 51.2 ^a,**^ | 228.30 ± 51.36 ^a,**^ | 235.50 ± 53.91 | 237.70 ± 49.62 |

Values are expressed as the mean ± standard deviation

^a^ Wilcoxon rank-sum test was tested for levels of red blood cell lead and plasma selenium between occasional or frequent alcohol consumer and non-drinkers.

^**^*p* < 0.01
